# Innovation, respiration and drug paraphernalia policy: a mixed methods study of crack pipe practice and respiratory harm in England

**DOI:** 10.1186/s12954-026-01470-6

**Published:** 2026-05-09

**Authors:** Magdalena Harris, Cedomir Vuckovic, Caitlynne McGaff, Casey Sharpe, Sujit Rathod, Alexandre Piot, Mat Southwell, Jenny Scott, Vivian Hope, Lucy Platt

**Affiliations:** 1 Department for Public Health Environments and Society, London School of Hygiene and Tropical Medicine, London, WC1H 9SH UK; 2 Department of Population Health, London School of Hygiene and Tropical Medicine, London, WC1H 9SH UK; 3 Coact, Bath, UK; 4 Centre for Academic Primary Care, Bristol Medical School, Canynge Hall, University of Bristol, Bristol, BS8 2PS UK; 5 Public Health Institute / School of Public and Allied Health, Liverpool John Moores University, 3rd Floor Exchange Station, Tithebarn Street, Liverpool, L2 2QP UK

**Keywords:** Crack cocaine, Harm reduction, Respiratory diseases, Drug legislation, Drug paraphernalia

## Abstract

**Background:**

Provision of equipment for the purpose of stimulant inhalation is prohibited under UK law. Crack cocaine use is prevalent and rising in England, where the SIPP (safer inhalation pipe provision) study piloted and evaluated a crack equipment and workforce training intervention. We report mixed method findings from the baseline component of the study, prior to intervention implementation. The aim of this paper is to situate quantitative findings regarding respiratory risk through qualitative exploration of crack inhalation practice in a context of stimulant equipment prohibition.

**Methods:**

In this paper we report descriptive findings from baseline survey data (*n* = 727) with a focus on thematic analysis of interview data (*n* = 33). Participants were recruited through drug treatment services and peer networks in six geographical locations in England, with survey eligibility criteria including crack use (injecting or smoking) in the past 28 days.

**Findings:**

Of the 733 participants who completed the baseline questionnaire, 727 (99%) smoked crack in the past month and were included for further analysis. A minority reported crack injection (28%). Over half (55%) used crack daily in the past month. Many (60%) reported respiratory symptoms, with one third experiencing a respiratory-related hospitalisation. Qualitative data illustrate ingenuity of crack equipment practice, with participants fashioning pipes out of materials ranging from umbrella handles, through to tin cans and plastic inhaler cases. Ash and stainless-steel scourer were primarily used as suspension materials. Accounts of practice illustrate connections between materials used and respiratory harms, the latter exacerbated by poor living conditions and limited availability of supports.

**Conclusion:**

We evidence a high burden of respiratory harm among people who smoke crack in England. Stimulant inhalation equipment prohibition contributes to a constellation of respiratory risk, with use of unsafe materials and impoverished living environments exacerbating health harms among a highly marginalised population. There is a need to reorientate drug services to support safer crack inhalation practice and reduce respiratory risk. Legislative change can facilitate this process.

**Trial registration:**

ISRCTN12541454 10.1186/ISRCTN12541454.

## Background

Harm reduction policy and practice traditionally orientate toward reducing harms associated with opioid use and injecting practice, such as blood borne virus (BBV) transmission and overdose fatality [1–3]. This paper sits alongside growing calls for the expansion of harm reduction programmes to attend to the distinct needs of people who use stimulants, particularly via inhalation [1, 4–6]. In England cocaine is the second most used illicit drug (after cannabis) [7] with 176,752 people estimated to use the base form of cocaine hydrochloride known as ‘crack’ in the year 2019–20; a 22% increase from 2016/17 [8]. Crack cocaine use is highly stigmatised and most visible among those experiencing housing, income and food insecurity [9–11]. Among socially marginalised people who use drugs, stimulants are often smoked or injected rather than snorted or taken orally. These modes of administration enhance intensity of effect but confer unique health risks, particularly when the equipment used is shared, repurposed or otherwise unsafe.

In this paper we focus on crack cocaine use in England, specifically in the context of safer inhalation equipment prohibition. Here, drug paraphernalia supply is regulated by Section 9A of the Misuse of Drugs Act 1971, under which it is a criminal offence to supply or offer to supply an object for administering or preparing a controlled drug, unless listed by exemption [12]. Section 9A was enacted in 1986 to allow for the provision of needles and syringes in response to the HIV epidemic, with amendments in 2003 in response to evidence of hepatitis C virus (HCV) transmission through sharing of ancillary injecting equipment (swabs, cookers, filters, water). Citric acid and vitamin C, as acidifiers for heroin injection, were included as exempt in 2003 and 2005 respectively, after lobbying from health care providers highlighted harms from use of unsafe alternatives, such as lemon juice-related candida outbreaks [13, 14]. The first and only exemption for inhalation equipment was in 2014, when—after sustained advocacy—foil for heroin smoking was permitted with the aim of reducing heroin injection and associated HCV transmission [15]. Provision of equipment for safer stimulant inhalation remains prohibited. Glass and metal pipes can, however, be sold for smoking legal substances or as ornaments, which allows for the sale of paraphernalia by private shops but inhibits provision through harm reduction orientated services or in combination with harm reduction information [16, 17].

Best practice guidance for the provision of safer crack inhalation equipment highlights that risk reduction is achieved through a system of components, not a single object [18, 19]. Recommended provision comprises a kit containing a heat-resistant borosilicate straight stem glass pipe, metal (steel or mesh) filters, rubber mouthpieces and push sticks for cleaning pipes and collecting crack residue. Ancillary equipment can include lip salves, chewing gum, lighters, condoms and lube [18]. In many European countries, legislation does not explicitly prohibit the provision of safer inhalation materials, and some which do, offer exceptions for harm reduction purposes. This has resulted in the legal provision of safer inhalation equipment in Italy [20], France [21], Estonia [22], Slovakia [23], Czechia [24], Slovenia[25], Austria [26], Belgium [27], Germany[28], Netherlands [29], Switzerland [30], Portugal [31], Spain [32] and Moldova [33, 34]. Similarly, in Canada, legislation does not explicitly ban or permit the possession of drug paraphernalia [35], but local municipalities impose differing levels of control. The distribution of stimulant inhalation equipment smoking is widespread in Canada, specifically in British Columbia [9, 36], Quebec [37], and Ontario [38]. In Latin America, stimulant inhalation kits are provided in Brazil [34, 39], with a temporary pilot in Mexico reducing sharing and use of unsafe pipe materials [40]. In both countries, the provision of drug paraphernalia, unless exempt, is illegal [41, 42].

Globally, provision of equipment for stimulant inhalation is uneven and challenging to map. Laws tend to allow for the distribution of drug paraphernalia unless items are specifically excluded. Countries governed by state and federal law may have pockets of state provision but no overarching federal approval, as in North America [43, 44]. Equipment provision may not be explicitly banned but precluded due to other considerations, such as perceived need and funding priorities. Community initiated operations can also precede and often inform programmatic provision. These ‘ground up’ initiatives may not be documented at the time, particularly if operating at the edges of the law. The Safer Crack Use Coalition of Toronto, for example, initiated crack inhalation equipment distribution in 2000, five years prior to the Toronto city government formally approving provision [45, 46]. This formalisation of grass roots-initiated equipment provision for people who use crack mirrors the introduction of needle and syringe programmes (NSP) following acts of civil disobedience by community activists and professional allies at the height of the HIV/AIDs epidemic [47–49].

As with early NSP implementation, crack inhalation equipment programs are controversial, inconsistently implemented and vulnerable to political whim and media fuelled ‘moral panic’. In 2007 political pressure following public controversy led to the defunding and cessation of the 2005 Ottawa crack inhalation equipment program, and in 2022, headlines such as “Uproar over ‘crack pipes’ puts Biden strategy at risk” dominated North American news after the announcement of a £30million federal grant to harm reduction services [50, 51]. The variety of supports to be funded, of which safer inhalation kits were but a fraction, was lost in the media furore over public spending on ‘crack pipes’, putting the political expediency of the whole grant into question. As a result, it was stated that any kits supplied were not to include pipes, undermining the HCV transmission remit which underpins many safer inhalation provision programmes.

Prevention of infectious disease transmission, by encouraging injection route transitions and reducing equipment sharing, is a politically viable rationale for inhalation equipment interventions. As with NSP, advocacy has capitalised on this concern. The Ottawa programme, for example, drew on “scientific evidence” of “potential for HCV and HIV transmission” through “multi-person crack pipe use” to achieve approval and funding [38, p.255]. Evaluation measures followed suit, focusing on HCV and HIV risk practices, particularly pipe sharing [38]. A year after the program was defunded, despite positive evaluation outcomes, Canadian scientists published biological evidence of HCV transmission through shared crack pipe use [52]. This strengthened advocacy for programme implementation [19] and bolstered a research focus on crack pipe sharing as a HCV risk practice. Surveys, including those initiated by community organisations [53], tend therefore to focus on measures of crack pipe sharing frequency and blood borne virus diagnosis [38, 54–56], with a qualitative literature providing insight into the conditions informing pipe sharing—including social and economic relations of exchange; environments of pipe scarcity; police presence and rushed semi-public drug consumption [9, 11, 37, 39, 57].

Of note in many of these studies is the absence of enquiry into or specificity about the *types* of pipes used for crack pipe smoking, even in conditions of crack pipe scarcity, including prior to safer crack pipe programme implementation. Where it is provided, information pertaining to the materiality of pipes used is generally sparse, mentioned in passing and/or indicates limited variety in equipment availability and preference. In a study of crack-pipe sharing among street-involved youth in Vancouver, for example, it is reported that only 5% of participants acquired their pipes from a health service, yet no primary data is provided on the alternative equipment in use. The authors suggest, drawing on prior studies, that this comprises ‘store brought’ ‘low quality’ pipes of unspecified material and form [55]. Other North American literature indicates that these may be glass pipes, with no mention of other materials used in the absence of service provision. As Boyd and colleagues note, in the absence of ‘quality’ heat resistant borosilicate pipes, ‘cracked and split’ glass pipes are ‘repeatedly used’ causing cuts on hands and lips and risk of ‘infection’ [57].

Where homemade materials and risk are mentioned this is primarily in relation to risk of cuts and burns, and linked association with blood borne virus transmission, as Ivsins et al. detail: “Crack users often use and share pipes made of various makeshift materials, including broken glass pipes, metal tubing, aluminium cans, car antennas, or glass ginseng bottles, all of which can cause cuts, sores, burns, and blisters in and around the user’s mouth” [11]. Two decades earlier Porter and Bonella [58] alerted of the risks of makeshift pipe use, after concerns voiced to them by people who used crack about HIV transmission potential through burns caused by metal pipes. In Briggs’ compelling visual ethnography with people who use crack cocaine in London, photographs provide insight into the components of a homemade pipe [59]. The pipe pictured is made from a small bottle, apparently hard plastic, with a syringe barrel inserted as a mouthpiece. Foil secured over the mouth of the bottle, punctured with holes, acts to hold a suspension device (in this case, likely ash) for the crack to melt into and vaporise once heated. These details, not present in the text, are inferred. ‘The crack pipe’, as referred to in a variety of contexts, here operate as a stable signifier of practice rather than a specific material and malleable object.

Our attention to the materiality of crack pipes occurred in the context of unsolicited reports of respiratory symptoms and makeshift pipe use among participants in a prior injecting-orientated study [60]. High prevalence of respiratory complications and conditions such as chronic pulmonary obstructive disease (COPD) among people who smoke heroin and/or crack are well evidenced [61–64]. Reference to pipe materials and respiratory harm are, however, largely confined to a case study literature documenting presentations for aspiration of crack pipe gauzes, fragments and even whole pipes [65–68], such in the playfully titled ‘foreign body aspiration getting weird’ in which a metal pipe is extracted from the owners lung [69].

In this paper we aim to reorientate attention from infectious disease transmission to respiratory risk, through presentation of mixed method findings generated with people who smoke crack cocaine in England. We situate quantitative data alongside a qualitative exploration of crack inhalation practice in a context of stimulant equipment prohibition. In doing so, we highlight the ways in which repurposed and homemade pipes can generate harms irrespective of and not reducible to sharing practice.

## Methods

The SIPP (‘Safe Inhalation Pipe Provision’) study piloted and evaluated a safer crack inhalation equipment and harm reduction training intervention. This was implemented for six months at three specialist drug treatment services, a sex worker service and two peer networks at three geographical locations in England, with local police approvals. A before and after cross-sectional survey was undertaken at these sites and three non-equivalent controls. The latter sites were selected based on geographical similarity but did not implement the SIPP intervention during the study period. Qualitative interviews and ethnographic observations were generated throughout the study duration. Study rationale, intervention and methods detail are published [70]. Here we outline methods specific to this paper, which reports select findings from the baseline component of the study, which includes a pre-intervention survey component and qualitative interview data generated prior to and in the early intervention period.

### Sample and recruitment

Participants were eligible to take part in the survey if they had used crack in the past 28 days, were aged 18 years or over, had capacity to consent and were recruited through a study site service or peer researcher. The SIPP team generated a random sample for the services to recruit from, based on client lists. This list was used for the first eight weeks of recruitment (primarily through telephone contact), after which recruitment was opened to all eligible service users who presented in person at the sites. This expansion was in response to challenges faced in contacting the randomly generated sample by phone within a short time frame, with the move to a convenience based sample reflecting real-world patterns of service engagement. In addition, peer-researchers recruited a sample quota through peer networks and street-based outreach. Peer researchers were individuals with current or former experience of crack use who were familiar with the local contextual crack market dynamics. They included volunteer staff from the Hepatitis C Trust, workers and clients at study sites, a peer-led technical support organisation, and non-affiliated community members employed by the study. Support and training on recruitment procedures, informed consent, confidentiality, and questionnaire administration were provided to peers by the research team. A total of 733 participants were recruited for the baseline survey, 590 through services and 143 through peer researchers.

Qualitative interview participants were recruited through the participating services and peer researchers. Participants were eligible if they had experience of using crack, were aged 18 or over and had capacity to consent. Participants were primarily recruited through the intervention sites, with purposive sampling for variation in gender, then age and ethnicity if possible. People with current experience of using crack were prioritised, with two participants who previously used crack recruited for their extensive experience and knowledge of local crack use practices and drug market dynamics.

### Survey data generation

The survey instrument was developed by the team, drawing on measures from comparable surveys [38, 53–55, 71] refined with service provider and peer input, and took approximately 30 min to complete. Questions covered demographics; crack and other drug use practices; health symptoms, diagnoses and hospitalisation; incarceration and policing experience; service access and income generation. The questionnaire was administered on tablet devices using ODK software [72] in English, with training provided to providers and peers at each study site on recruitment methods, informed consent, and questionnaire administration. Participants were recruited through three intervention drug treatment services and one sex worker service, three comparison site drug treatment services and peer outreach at two intervention and one comparison site. Data collection took place between March and October 2023, with most (all intervention sites) completed prior to SIPP intervention implementation in July 2023. The questionnaire was primarily administered by service providers and peers, with an option for participants to self-complete if they preferred. Questionnaire data were encrypted immediately upon completion, uploaded via the internet to a secure server administered by the lead University, and automatically deleted from the tablet device.

### Qualitative data generation

In-depth interviews analysed for this paper were conducted by either CV or MH between May and December 2023. Interviews were informed by a topic guide exploring local context and drug market dynamics; personal living circumstances; crack use practices; equipment use and preferences; risk perceptions and health issues; service access and engagement; and perceptions of the SIPP intervention. Participants who smoked crack were asked if they had a pipe on them and, if consenting, had their pipe photographed. Photographs were taken with a mobile phone camera and did not include any identifiable body parts or information. Interviews mostly took place in a private room at the participating service, with some in participants homes, others in an open area of a sex worker drop-in service and one in a park. Interviews varied in duration dependent on circumstances and participant priorities, mean 35 min. Interviews were audio recorded, transcribed verbatim by a professional transcriber with whom the team have a confidentiality agreement, checked and anonymised by CV or MH. Both researchers generated detailed ethnographic field notes for each site visit and interview encounter, with a focus on local contextual and social dynamics.

### Analysis

After excluding six survey participants who did not smoke but exclusively injected crack, we analysed questionnaire data on demographic characteristics, drug use, crack smoking practices, and respiratory health. Percentages are reported for categorical measures. Analyses were conducted using StataSE 18.

Qualitative analysis was undertaken by MH and CV and informed by principles of constructivist grounded theory which emphasise early and ongoing inductive analysis with movement towards conceptual categorisation and mid-range theory development [73]. Initial coding involved line-by-line open coding of a subset of transcripts using process-oriented gerunds (e.g. “fashioning pipes”, “negotiating risk”). These were consolidated to inform a focused coding framework, comprising ‘first level’ codes or categories. All transcripts were coded against the coding frame in NVivo [74]. Second-stage coding comprised inductive open coding of data under each category, comparison and categorisation to inform analytic interpretation and theme development. Analysis of primary codes pertaining to pipe equipment and health harms inform this paper. Analytic memos were generated throughout, building connections between, for example, material pipe practices, respiratory harm, and structural context. While grounded theory principles informed analytic procedures, the aim was not to generate a formal grounded theory. Rather, focused coding and category refinement informed an inductive thematic analysis. Themes were developed iteratively through discussion between authors and in dialogue with survey findings to support mixed-method integration.

Photographs of participant pipes are included for illustrative purposes to support reader understanding of pipe construction and materiality; they were not analysed as a discrete visual dataset.

### Ethics

Ethical approval was awarded by the London School of Hygiene & Tropical Medicine (Observational/Intervention Research Ethics Committee [28102]. All participants were provided with a detailed participant information sheet, a verbal summary of the project and the opportunity to ask questions prior to providing written consent. They received £10 cash in renumeration for survey participation and £20 for interview participation. All participant names in this paper are pseudonyms.

## Results

### Baseline survey data: highlighting respiratory risk

In total 733 people completed the baseline survey, of whom 727 had smoked crack in the past 28 days and are included in analysis. Participant demographics, criminal justice involvement, drug use practices and self-reported health conditions are reported in Table 1. Crack use was frequent and intensive: 55% reported daily use in the past 28 days, and 29% both smoked and injected crack. Use of homemade or repurposed pipes was common (69%), alongside use of store-purchased glass or metal pipes (52%). Stainless steel scourer or metal gauze was used by 73% of participants as a suspension device, and 42% reported using ash. Respiratory burden was substantial. Sixty percent reported at least one respiratory symptom in the past 28 days, including breathing difficulties (40%), coughing phlegm or blood (35%), and chest pain (31%). One third reported hospitalisation for a respiratory condition, 31% an asthma diagnosis, and 12% a diagnosis of COPD or emphysema. Cuts or burns to the hands or mouth were reported by 49%.

**Table 1:** Sample characteristics, drug use practices and respiratory conditions

		*n* = 727
Gender	Cis male	508 (70%)
	Cis female	207 (28%)
	Trans, non-binary or declined to answer	12 (2%)
Ethnicity	White	630 (87%)
	Black/Asian/other minoritized ethnicity	97 (13%)
Age	Mean	42 years
	Range	19–72 years
Accommodation (past year)	Stable (flat/house—solo or shared)	392 (54%)
	Unstable (hostel, sofa surfing, prison)	205 (28%)
	Street homeless	130 (18%)
Criminal justice involvement	Imprisoned (ever)	513 (71%)
	Stop and search (past 6 months)	278 (38%)
Opioid use and agonist treatment	Heroin use (past 28 days)	564 (78%)
	Opioid agonist treatment	446 (61%)
Crack use practice (past 28 days)	Inhalation/smoking only	513 (71%)
	Inhalation and injection	214 (29%)
	Daily crack use	400 (55%)
	Daily crack inhalation	393 (54%)
Inhalation equipment (past 28 days)	Homemade/repurposed pipe use	502 (69%)
	Store brought pipes: metal or glass	259 (52%)
	Glass miniature spirit bottles	131 (26%)
	Plastic inhaler cases	119 (24%)
	Tin cans	106 (21%)
	Stainless steel scourer	367 (73%)
	Ash	211 (42%)
Respiratory symptoms (past 28 days)	Breathing problems	317 (40%)
	Coughing phlegm or blood	252 (35%)
	Chest pain	228 (31%)
Respiratory diagnoses (ever)	Asthma	222 (31%)
	COPD or emphysema	84 (12%)
Respiratory-related hospitalisation (ever)	Pneumonia or bronchitis	138 (19%)
	Asthma, COPD or lung cancer	111 (15%)

These findings highlight respiratory risk as a significant concern. Understanding how smoking practices contribute to these harms requires attention to the materiality of pipe construction and use, to which we now turn.

### Qualitative data: understanding practice

Of the 33 participants interviewed, 14 identified as female and 19 male, the majority as white British (*n* = 30) and, excluding 4 missing ages, their average age was 44 years (range 25–57). Ten interviews were conducted prior to SIPP implementation, with 23 in the months following. The focus here is on crack inhalation practice and experienced health impacts prior to acquisition of a SIPP pipe. This temporal focus aligns with the baseline survey data with analysis orientated to unpacking issues reported therein. We present findings against two themes, attending to material pipe practices and embodied health experiences.

### Innovation and transition: material pipe practices

Participants were asked to describe the types of pipes and suspension devices they used, the materials and methods used to make pipes, their equipment preferences and if their practice had changed over time. Understanding practice requires awareness of pipe components and their respective purposes. Crack pipes generally comprise a chamber, with one opening for heating and the other for inhalation. The crack is placed on a suspension device (eg. ash or stainless steel scourer), heated, melted and vapourised. Residue (or ‘recycle’) may accumulate inside the chamber and later be scraped or washed out with ethanol and reused. Participant preferences reflected considerations of durability, concealability, crack delivery efficiency, residue collection, cost, and risk of burns or cuts.

Most participants, when asked, had a pipe on their person. All who did so, were happy to for this to be viewed and photographed. These encounters illustrated how, for some, the pipe operated as an extension of the self, through continual carriage but also through expression of individual preference, skill and innovation in their fashioning:

I use a glass bottle

**Ah yeah. Have you got it on you?**

Yeah.

**Can I have a look?**

Don’t go anywhere without my pipe

**Oh nice. Did you make that yourself?**

Yeah. With holes at the bottom.

**That’s lovely.**

It’s a bit empty at the minute. *[Laughter]*

**Is it your preference to have a pipe like this?**

Yeah. I don’t enjoy smoking on any other pipe.

**What do you like about this pipe?**

I don’t know, I just get my hit better off it. (Jada, Nottingham)

**Fig. 1:**
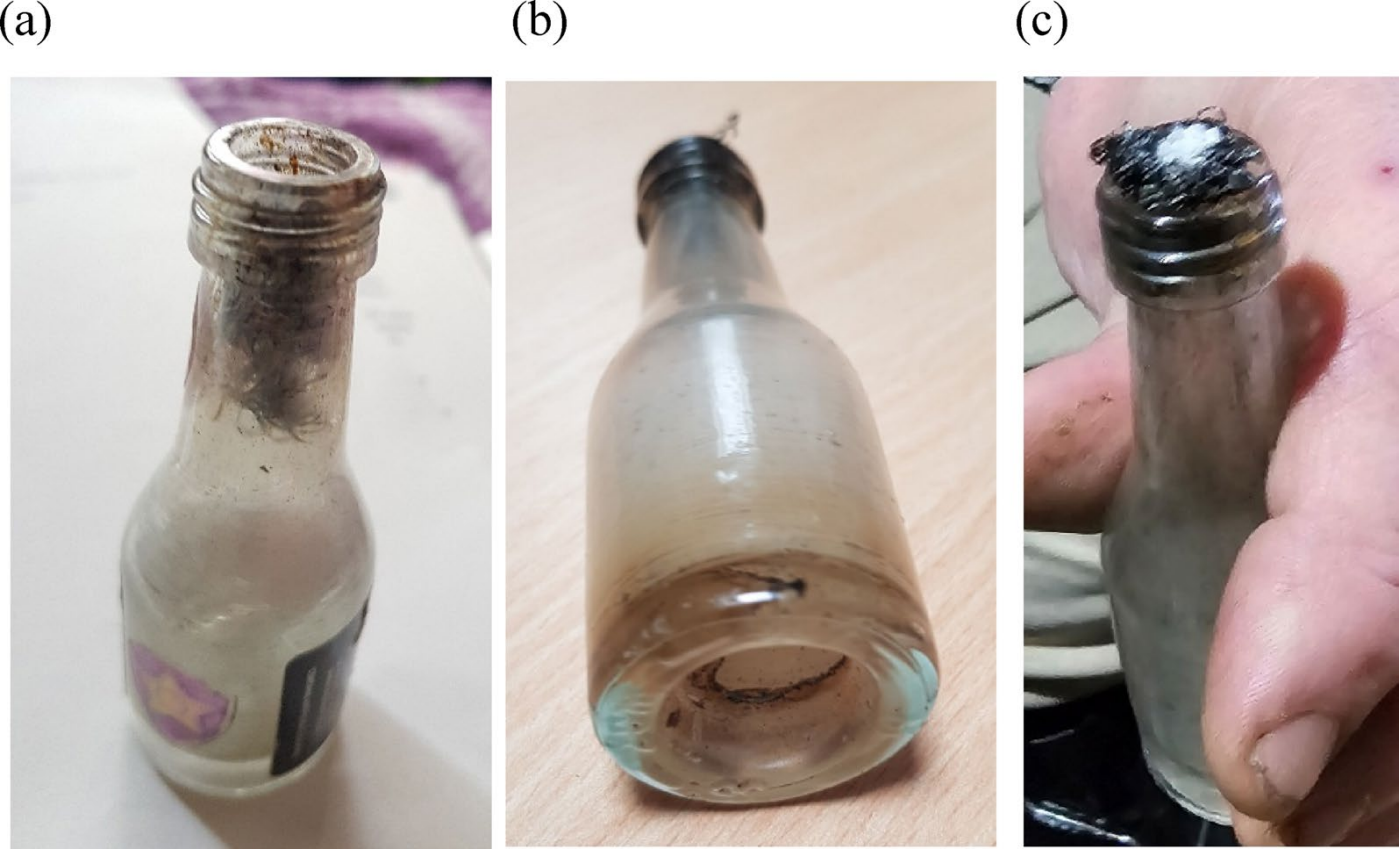
Glass bottle pipes, illustrating **a** stainless steel scourer in bottle neck; **b** mouthpiece at bottle base; **c** crack sitting on suspension device (stainless steel scourer)

A crack pipe can be quickly fashioned from everyday materials such as a plastic bottle, plastic asthma inhaler, or tin can (for the chamber), tin foil, rubber band, cigarette ash or stainless steel scourer (for suspension device) and pen chamber or roll of tinfoil (for the mouth piece). In the excerpt above, Jada refers to use of a glass bottle. These are typically miniature spirit bottles with a hole made in the base as the mouthpiece and a penny-sized ball of stainless-steel scourer or ‘wire wool’ inserted in the bottle neck to hold the crack (see Fig. 1).

Participant accounts highlight dexterity in innovating and adapting when their preferred equipment was not to hand. As Ollie (Nottingham) says: “Anything you can think of, I’ve tried to make a crack pipe out of”. He details the transformation of umbrella handle to pipe:

So the umbrella, in the middle of the tube where you like, where you close it and it’s hollow, so I’ve had to like bend the umbrella like that and snap it off and then bend it on the other side and snap it off and then take a fork and … because they were flat on the ends, open that out and put the gauze in there and smoke it like that.

Radiator fittings, disposable vapes, naloxone syringes, perfume bottles and pram handles all feature in participant accounts of pipe manufacture, generally as last resort rather than preferred options.

**Fig. 2:**
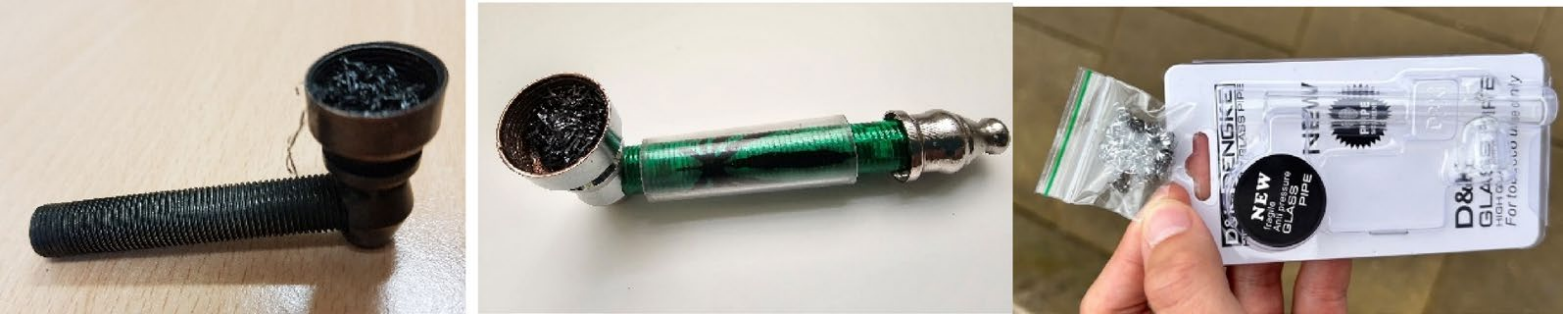
Metal pipes with stainless steel scourer, purchased glass pipe provided with scourer

Participants preferred, or most commonly carried equipment, was a small metal pipe available for purchase from newsagents and other stores, generally associated with cannabis consumption. These ‘weed pipes’ comprised a short metal bolt/tube with a metal bowl attachment, with glass stemmed bowl pipes also available, but less popular (see Fig. 2):

I use a gauze pipe, I just smoke it, so basically I use either a glass or a metal tube, with wire wool in the end … you can buy them in shops little metal kind of tubes, you screw on a bulb on the end … apart from it’s not got the kind of mesh bit on it, it’s just got wire wool, like a scouring pad. (Ken, Bristol)

As Ken emphasises, these pipes are repurposed for crack use by replacing the accompanying wire mesh filter with a ball of stainless-steel scourer or ‘wire wool’, or in some cases, with the addition of ash. This provides the density required to hold and contain the crack as it is smoked. At two sites, participants spoke of being supplied a small bag of stainless-steel scourer with the pipes, in tacit acknowledgement of the purpose to which they were to be used:

I’d get a glass one [pipe] from the shops on [street], they all sell them … [I’ll ask for] some gauze, they give you like a round, like a lump of it, for a quid. (Tracey, Bristol)

When you buy a pipe down in X [Shop] they give you a little ball of wire wool to put in it.

**Yeah, they sell it to you or…?**

No, no, just get it with it.” (Scott, Mansfield)

For many, arriving at the use of a metal pipe (or their preferred alternative—for some, the glass bottle), was informed by a prior trajectory of exploration and innovation, in which preferences were solidified and some materials and modes of smoking rejected as inefficient and/or unpleasant. Brandon (Bristol) outlines a common trajectory:

So the first time I used a can, a can with ash on, and then after that we moved quickly onto an inhaler, like a blue inhaler with some tin foil and then ash on that, and we stuck with those for a long time, and it wasn't until a lot later that we got into glass stems.

This is mirrored by Neil, also from Bristol:

When we first started it was just like use a can, a metal can, that was the go-to thing back then, and then it was use the plastic inhalers and then like if you had a house to smoke from you’d make yourself a bottle, like a nice bottle like that filled with like some Cherryade or something … And then when you get like really bad you’d have your own little personal pipe that you’d have in your pocket, like little metal one… when you get like to your lowest like you’ve always got a pipe with you, d’you know what I mean, just in case.

For Neil, the personal pipe, always kept close, signified the latter stage of crack use: “when you get to your lowest”. This account may reflect Neil’s distance from his use, having ceased crack consumption six months prior to the interview, but also illustrates how a change in pipe materials can mirror increased frequency and intensity of use. When increased use necessitates keeping a pipe on the person, for example, concerns about equipment durability, longevity, efficiency and compactness become more important.

Tin cans are none of these things. They offer, however, a quick and relatively easy smoking device, particularly if limited to street-based materials. One participant demonstrates transformation of coke can to pipe in less than one minute, another describes the process:

Uh, well rip the tip off, you know, the ring pull, snap it so it’s like a little spike on the end of it, bend the end of the can, not the mouth bit but the other end so it’s like flat, and then just poke the holes and make a shotgun hole at the side and then *[makes sucking noise]* and then let go. (Darren, Nottingham)

Neil (Bristol) contrasts cans with inhalers, stressing ease of adaptability:

Like obviously like the inhalers like you just put a bit of foil over the top like with elastic band, but with a can you don’t need elastic band, you don’t need anything, so that’s why everyone makes the pipes out of cans.

**Fig. 3:**
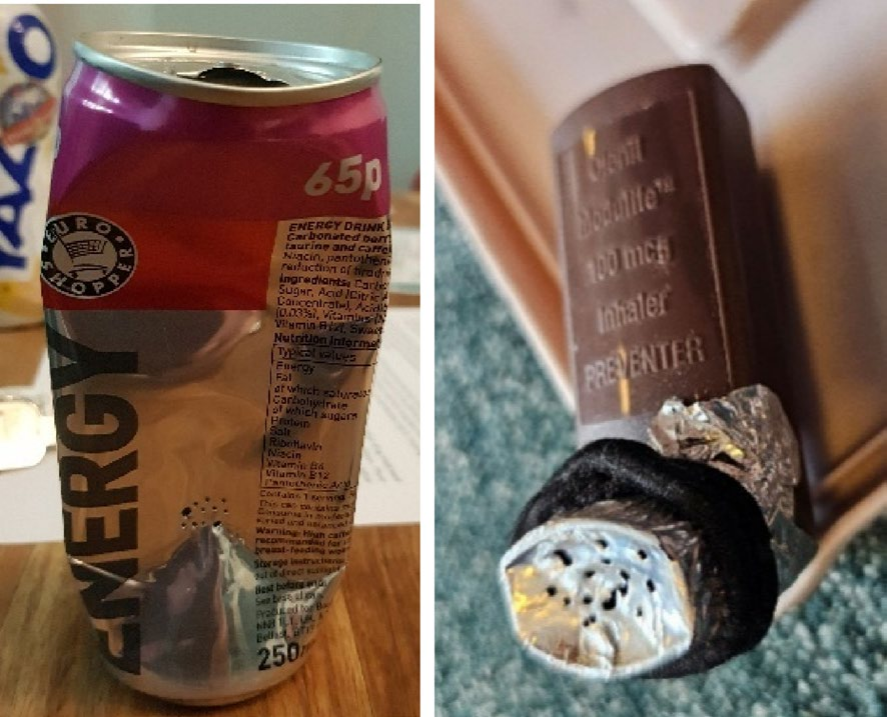
Can pipe, inhaler pipe. Ash is placed on holes pierced in can and foil

Unusual in this statement is the present tense of ‘everyone makes’, with most participants referring to use of tin cans as a past activity, a rite of passage almost, until moving onto better things. Brandon refers to plastic inhalers, glass bottles and plastic methadone bottles as: “all pretty much better than the can, basically, which was very sort of ghetto and didn't work all that well, but it did the job at the time.” See Fig. 3 for illustration of inhaler and tin can pipes.

Tin cans are traditionally used with ash to hold the crack when smoked. Ash is not always easy to get hold of, with participants variously speaking of: breaking into cars to empty ashtrays; taking ashtrays from pubs or cigarette butts from rubbish bins; being on ‘smoking duty’ when crack was to be consumed in a group; and the ongoing expense of tobacco purchase:

Well you have to buy cigarettes ... you burn all the cigarette down and put the ash on it … [gauze is] cheaper and easier … when you melt it on the gauze, you can melt it on and it's still there and you can keep smoking it, whereas when it's on the ash, once you've lit it it's gone. (Dawn, Nottingham)

This narrative reflects the way in which trajectories of use combine assessment of the relative strengths and weaknesses not only of the pipe body or chamber, but the suspension devices most suited to its use. Movement away from chambers used with ash (cans, inhalers, plastic bottle bongs) towards those which afforded use of stainless steel scrubber or ‘gauze’ (miniature glass bottles, metal and glass stemmed pipes) aligned with movement towards the smaller, more portable and durable materials as expertise, expectation and, often frequency of use, also increased.

Of note are regional differences in preference, with participants in one site more frequently reporting use of ash, including in metal ‘weed pipes’, whereby the thin metal gauzes supplied with the pipes were retained and ash placed on top of these to provide a bed or suspension device for the crack to sit on:

When you buy them pipes you get like, you already get gauze in it but they give you another ten in a little packet and they're little round gauzes … you put one in the dip and then put your ash and the crack on top yeah. (Sam, Coventry)

This site where ash was predominately used stood in stark contrast to others in which stainless-steel scourer was used in ‘weed pipes’ and ash generally spoken of as a thing of the past:

Oh no, they don’t use them no more, no one uses that [ash]. No, everyone used to, ah that takes me back some years now … inhaler with ash on, bloody hell yeah. (Dale, Mansfield)

Accounts by participants in one semi-rural market town indicated regional differences more broadly, with Scott (Mansfield) referring to practices in another small town 30 min away ‘up the train track’: “It’s like if you go just like up the train track a bit into [neighbouring town] and that, they don’t use nothing but ash…. Yeah, [we] don’t use ash, we use wire wool”.

Expertise was demonstrated not only through the mechanics of pipe fashioning, but the preparation of the suspension device for holding the crack. Not all ash is equal as evident in participant accounts:

Just use virgin ash for this, when it burns down, put it straight on…. If you use like bad [ash], it’s not as, it doesn’t absorb properly…Bad ash, it’s gone like powder and powdery, it’s got to be like virgin ash off the fag. (Stephen, Coventry)

In extremis, participants recounted trying ash from other sources, such as newspaper, but found the consistency too fine for purpose. As with ash, discernment was required in stainless steel scourer acquisition, with most participants who used this demarcating ‘good’ from ‘bad’ scourer:

I only use a certain brand of it because a lot of them, they taste horrible and like it sounds like fireworks go off them and they taste horrible (Hannah, Nottingham)

There is like two sets of wire wool, one’s crap and horrible but the other’s standard. … It just tastes foul, and it disintegrates quicker as well, the other one. (Darren, Nottingham)

Preparing the stainless-steel scourer involved burning it with a flame prior to use, an almost ubiquitous learned practice reported by participants:

Yeah, you have to burn it. Put it on a pen or something, burn the ends, burn off the black stuff, and then put it in the bowl, don’t put it in sterile because that gives you a bit of choke. (Alison, Nottingham)

The wire wool, which is just like scouring pad, I take it, and I burn it so burn any of the kind of chemicals and stuff that’s on that, you kind of see smoke coming off so it’s cleaner. (Ken, Bristol)

These accounts indicate concern not only with the quality and experience of the crack hit, but the impact of this on their health, with efforts made to remove perceived chemical residues to obtain a ‘cleaner’ hit, but also to avoid ‘choke’. It is to these health impacts that we now turn.

### Inhalation and respiration: embodied health experiences

Participant narratives highlighted the way in which different pipes accord different health harms or risks. Purchased glass stem pipes, for example, were commonly referred to as fragile resulting in cuts to fingers or mouth from breakages and sharp edges. The popular metal ‘weed pipe’ could become very hot, with burns to hands and lips noted by many: “I burn my mouth all the time” (Alison); “it gets really hot really quick … my fingers are like calloused now because of how much I’ve burnt them (Ollie).” One participant referred to heating his metal pipe to such a degree that ‘the aluminium started to melt’ but few extrapolated from this to consider the heat of the vapour inhaled, as noted below:

With the little small metal ones they get really hot and the smoke can get hot and that burns your lips and burns your tongue (Neil, Bristol)

Here immediate proximal burns are noted—to the lips and tongue. Inhalation of hot vapour risks damage to the throat and lungs. This is not commented on, possibly because the numbing effect of cocaine can reduce awareness of pain or burning after inhalation and obscure acute respiratory impact.

With plastic inhaler use, respiratory harm was rendered more visible—specifically regarding the collection and consumption of crack residue or ‘recycle’ from the pipe chamber. Sharp metal implements can be seen as efficient tools to scrape out recycle, but they can damage pipes and result in shards of glass, metal or plastic being incorporated into the drug smoked. This process is generally imperceptible and not commented on by participants. Pipes made from inhalers provide a notable exception:

I’ve seen people scraping it with a knife and where they’ve scraped so hard there are blue curly bits from the inhaler plastic in with [the recycle], and like you can smell it when they burn it and it’s just like smoking plastic. (Neil, Bristol)

Yeah, had a lot of plastic in it. So it would be shavings of blue plastic mixed in with it, which wasn't very nice […] I did think about the long-term effects of inhaling so much plastic, but it was a thought that briefly came, and then went when I needed another hit again […] I had very severe like coughing problems, wheezing, coughed up a lot of phlegm. It definitely didn't help things, it was a pretty unhealthy way of going about doing it. (Brandon, Bristol)

The accounts above indicate a tension—between risk made visible (coloured shavings, potent fumes) and the value of recycle as a potent resource not to be wasted. In this way respiratory risk is incorporated as an almost inevitable part of the smoking process, held in the back of the mind while overridden by more pressing concerns. For Stephen (Coventry), this tension was captured in the materiality of recycle itself, a brown to black resinous substance catching hold of the pipe interior as it might the lungs:

Recycle, scrape it, pipe scrape, scrape all the, to get some more … Yeah, so and I’ll look at that and think, oh that’s going on me lungs, but it does, it doesn’t stop me though. … If that’s going, building up on the pipe, what’s it doing to me lungs?

The practice of smoking on scourer also required some care, with participants noting that if not replaced frequently the stainless steel could degrade, with fragments falling down the pipe to be inhaled with the crack vapour. As Darren states: “I’ve been picking wire wool out me gums for days after.” Accounts of entire gauzes being swallowed or large fragments burning the mouth were common:

Yeah, well if you’re using something like a steel wool or just some random gauze you got from a smoke shop or something … there’s a high chance that when you’re pulling on it it’s going to shoot down right into your mouth and burn your lips or inside your mouth, like I’ve had burns on like the back of my tongue from that stuff before and that’s horrible. (Callum, Mansfield)

I’ve swallowed the gauze many times because I suck me pipe … my pipe’s a metal one and I put the gauze in and I use like that and it sucked through the gauze and went “pujung” … down my throat man …. It burnt all my throat. I was dying, man. I was absolutely dying. (Emma, Nottingham)

Emphasised in both accounts is the velocity of this action—the gauze ‘shoots down’ into the mouth, goes ‘pujung’ down the throat—and an associated intensity of inhalation (‘pulling on it’, ‘sucked through’). Strong inhalations, often aiming for depth and retention of vapour, can in this way exacerbate respiratory risk—both from metal fragment inhalation but also retention of hot vapour.

A strong inhalation technique also appears to exacerbate risk when ash is used as the suspension device. Neil, who now uses scourer, refers to ash as “the worse thing’, as “the next morning when you get up after having a big sesh like you cough and it’s like coughing up pure ash.’ Stephen (Coventry), who has only ever smoked crack using ash, states:

I’ve had years and years I’ve had, oh in the morning when I’ve first cigarette, the amount of rubbish I bring up off me chest is unreal, you know, I’m struggling, coughing, really coughing and bringing a lot of rubbish up, I should go to the doctors with it really. … Just grey phlegm, grey. … I think it has got worse, since I started smoking crack, yeah. But my head will tell me anything but that.

Stephen, as with many participants, is a long-term tobacco smoker, and his account illustrates the ambiguity in attributing causality. Although in his late 50s, Stephen has only been regularly smoking crack for the past three years, a practice which has likely exacerbated but not solely caused the respiratory symptoms described above.

Ambiguity regarding causality recurred across accounts. When asked, many recalled experiences of breathlessness, coughing blood or grey/black phlegm, hospitalisation for pneumonia and diagnoses of respiratory conditions such as COPD or asthma:

I’ve had double pneumonia and all sorts like. But I’ve always had bad lungs…

**Tell me about getting pneumonia and stuff, do you think that was related to the crack use?**

Probably, but like I always get bronchitis in winter anyway, and just cold damp conditions, not looking after myself properly, probably too much smoking. I don’t know … like a culmination of stuff … the last time I got ill it was so bad I could barely breathe, walk, my partner ended up, it was Christmas, ended up taking me to a friend’s house, and I got put in the back living room basically and didn’t move off the couch for about four days. I was like was drowning in my own lungs. (Brandy, Nottingham)

Brandy expressed caution in drawing an explicit association between her crack smoking and poor respiratory health, emphasising the ‘always’ of her ‘bad lungs’ and ‘bronchitis in the winter’. Here difficult circumstances, such as living without adequate heating or ventilation, cumulate with strategies to cope in such circumstances—such as smoking crack—in illnesses so viscerally described above. There is an inevitability in such accounts, whereby poor respiratory health is familiar, incorporated, and medical care seen as a last resort or not mentioned at all.

Some participants did draw connections between their crack use and respiratory health, but this may have also been in response to the known focus of the study, and in anticipation of the forthcoming pipe provision intervention. Lindsey (Mansfield), for example, states: “Yeah, I’ve got that OCPD [COPD] thing … and I think that’s through not having the right pipes and stuff”. She describes her lungs as “terrible”, and how her respiratory and mental health intertwine to ill effect: “I just can’t breathe. And if I panic, I have panic attacks, I can’t get very far. I panic attack, I sort of go dizzy. I pass out”.

Ollie, also diagnosed with COPD, frames this in terms of underlying poor respiratory health, also emphasising the role crack plays in its exacerbation:

My lungs have deteriorated. I mean, I had COPD anyway and since I’ve started smoking crack again, it’s just, my lungs have deteriorated so quick … I can’t walk as far, I can’t get upstairs anymore, I’m coughing constant, constantly, like my voice keeps going. I know it’s having a very bad effect on me, so. (Ollie, Nottingham)

As participants spoke of trajectories of pipe use, so is health experienced as a trajectory—intertwining with the resumption or cessation of smoking practice as well as living circumstances and interactions with systems of medical care. Ollie voices the awareness which runs as an undercurrent in many participant accounts: “I know it is having a very bad effect on me”, but as with attributions of causality, putting this awareness into action is not straightforward. For many, crack use has—as with poor respiratory health—become incorporated into their day-to-day and, for some, continues to provide meaning and purpose.

## Discussion

This paper reports mixed method demographic, risk practice and respiratory health related data from a sample of people who primarily smoke crack cocaine in the UK. It addresses an important gap in the literature by exploring the material forms and practices associated with homemade and repurposed crack pipe use in a context of equipment prohibition. While a growing qualitative literature draws attention to the social relations and risk environments informing crack pipe sharing [37, 57], the specificity of what it is that is being shared is often not elaborated. By foregrounding the materiality of pipes and suspension devices, our analysis extends beyond sharing behaviours to examine how the physical properties of equipment, and the ways in which it is improvised, shape respiratory risk. Attention to equipment materiality in the context of crack smoking practice alerts not only to heterogeneity (of practice, equipment and risk), but the ingenuity of crack smoking practitioners fashioning equipment in contexts of scarcity and constraint. These constraints—legal, material, and socio-economic—intertwine with pipe craft and practice to create and exacerbate health harms, particularly those affecting respiratory health.

SIPP is a mixed-method study, with baseline survey data reporting a high burden of respiratory symptoms, diagnoses and hospitalisations among respondents from six different geographical locations in England. Qualitative data unpacks the story behind these figures, illustrating the multiple pathways through which homemade and repurposed pipe use can exacerbate respiratory harm. Participants reported widespread use of everyday objects such as tin cans, asthma inhalers, metal bolts, glass and plastic bottles. Fume and hot vapour inhalation, alongside cuts and burns from these materials are documented [10, 38, 53, 54, 75–78]. The materials used as suspension devices—commonly stainless-steel scourer or ash with tin foil—also introduce risks. Our findings are supported by a recent experimental study demonstrating significant structural changes and degradation of stainless steel scourer after manipulation and heating [79]. Despite attempts to reduce harm, such as burning stainless-steel scourer to remove chemical residue prior to use and using ‘new’ ash, accounts of burns, lacerations and ‘coughing up black’ were common. These visible harms, including burns to the mouth, were emphasised over those less tangible, where the numbing effects of cocaine may mask acute respiratory injury.

The conditions in which crack smoking practices occur—marked by criminalisation, stigma, insecure housing, and poverty—further compound respiratory harms. Respiratory conditions are the third leading cause of death in England, with mortality starkly stratified by income [80]. A entrenched retraction of the welfare state, from the 2010 implementation of austerity policies, has made access to safe secure housing and the ability to keep warm increasingly difficult for people on low incomes [81]. Participant accounts illustrate the burden of respiratory harm on daily life, with reports of limited mobility due to breathing difficulties common. While some clearly articulate an interplay between their crack use and declining respiratory health, for many attributions of causality are ambiguous. Pre-existing health issues, housing insecurity, cold and damp living conditions, and concurrent use of other smoked substances (such as tobacco) are highlighted by many as part of this constellation of respiratory risk.

These dynamics can be conceptualized as forms of “slow violence” [82]: harms that are gradual, invisible, and dispersed across time and space. Here policy prohibitions also enact a slow violence—a damage through absence, in which both the needed equipment and the population who use this is rendered ‘out of sight’. Prohibition against provision of smoking equipment for the purposes of crack use, necessitates use of makeshift and repurposed materials but also negates recognition of people who smoke crack at the services where equipment for other drug use is supplied. This in turn reduces opportunities for respiratory assessments and diagnosis, as well as the provision of other needed health and social supports. Provision of safer inhalation equipment offers a critical opportunity to engage a highly marginalized population with health and social services [9, 44, 57, 83]. This is relevant not only to people who use crack. Drug related deaths in England are now at the highest since recording began in 1993 [84], and the potential for underlying respiratory conditions to exacerbate overdose risk among people who use opioids should not be underplayed.

International experience offers important lessons. Evaluations from Canada and Ireland, where safer crack pipe distribution is permitted, demonstrate that providing safer inhalation equipment reduces the harms associated with homemade pipes—including burns, cuts, and respiratory injury—without increasing frequency of use [10, 53, 76, 85]. Widespread distribution also reduces the urgency of rushed, unsafe practices and can encourage transitions away from injecting, with significant public health benefits [38]. Our findings, however, caution that safer pipe interventions must account for strong user preferences and established smoking practices. Participants often expressed attachment to particular homemade pipes, highlighting preferences for portability, durability, and familiarity. Previous harm reduction efforts (e.g., low-dead-space syringes) demonstrate that transitions in equipment use are possible but require sensitivity to user expertise, practice trajectories, and motivations [86]. This is supported by Irish outreach initiatives in which pipe acceptability was crucial in fostering service engagement [83].

## Conclusion

Our findings highlight a high burden of respiratory symptoms and conditions among people who primarily smoke crack cocaine in England. In a context where provision of safer inhalation equipment is prohibited, makeshift pipes constructed from hazardous materials can not only cause acute injuries but contribute to longer-term health harms. Safer inhalation equipment provision offers a critical intervention point. When informed by and meeting client preferences, this can offer a gateway to broader health and social supports for a population largely excluded from care. In the context of record drug-related deaths and widening health inequalities in England, integrating safer inhalation equipment provision into policy and practice is not only evidence-based but an ethical imperative.

## Data Availability

The datasets generated and/or analysed during the current study are not publicly available due to study finalisation and data set archiving preparation but will be available from the corresponding author on reasonable request.

## Abbreviations

BBV: Blood borne virus
COPD: Chronic pulmonary obstructive disease
HCV: Hepatitis C virus
HIV: Human immunodeficiency virus
NSP: Needle and syringe programme
ODK: Open data kit
SIPP: Safe inhalation pipe provision
UK: United Kingdom
